# The intermediate in a nitrate-responsive ω-amidase pathway in plants may signal ammonium assimilation status

**DOI:** 10.1093/plphys/kiac501

**Published:** 2022-10-27

**Authors:** Pat J Unkefer, Thomas J Knight, Rodolfo A Martinez

**Affiliations:** Biosciences Division, Los Alamos National Laboratory, Los Alamos, New Mexico 87547, USA; Department of Biological Sciences, University of Southern Maine, Portland, Maine 04104, USA; Millennium Enterprises, Santa Fe, New Mexico 87547, USA

## Abstract

A metabolite of ammonium assimilation was previously theorized to be involved in the coordination of the overall nitrate response in plants. Here we show that 2-hydroxy-5-oxoproline, made by transamination of glutamine, the first product of ammonium assimilation, may be involved in signaling a plant’s ammonium assimilation status. In leaves, 2-hydroxy-5-oxoproline met four foundational requirements to be such a signal. First, when it was applied to foliage, enzyme activities of nitrate reduction and ammonium assimilation increased; the activities of key tricarboxylic acid cycle-associated enzymes that help to supply carbon skeletons for amino acid synthesis also increased. Second, its leaf pools increased as nitrate availability increased. Third, the pool size of its precursor, Gln, reflected ammonium assimilation rather than photorespiration. Fourth, it was widely conserved among monocots, dicots, legumes, and nonlegumes and in plants with C3 or C4 metabolism. Made directly from the first product of ammonium assimilation, 2-hydroxy-5-oxoproline acted as a nitrate uptake stimulant. When 2-hydroxy-5-oxoproline was provided to roots, the plant’s nitrate uptake rate approximately doubled. Plants exogenously provided with 2-hydroxy-5-oxoproline to either roots or leaves accumulated greater biomass. A model was constructed that included the proposed roles of 2-hydroxy-5-oxoproline as a signal molecule of ammonium assimilation status in leaves, as a stimulator of nitrate uptake by roots and nitrate downloading from the xylem. In summary, a glutamine metabolite made in the ω-amidase pathway stimulated nitrate uptake by roots and was likely to be a signal of ammonium assimilation status in leaves. A chemical synthesis method for 2-hydroxy-5-oxoproline was also developed.

## Introduction

**Figure 1:**
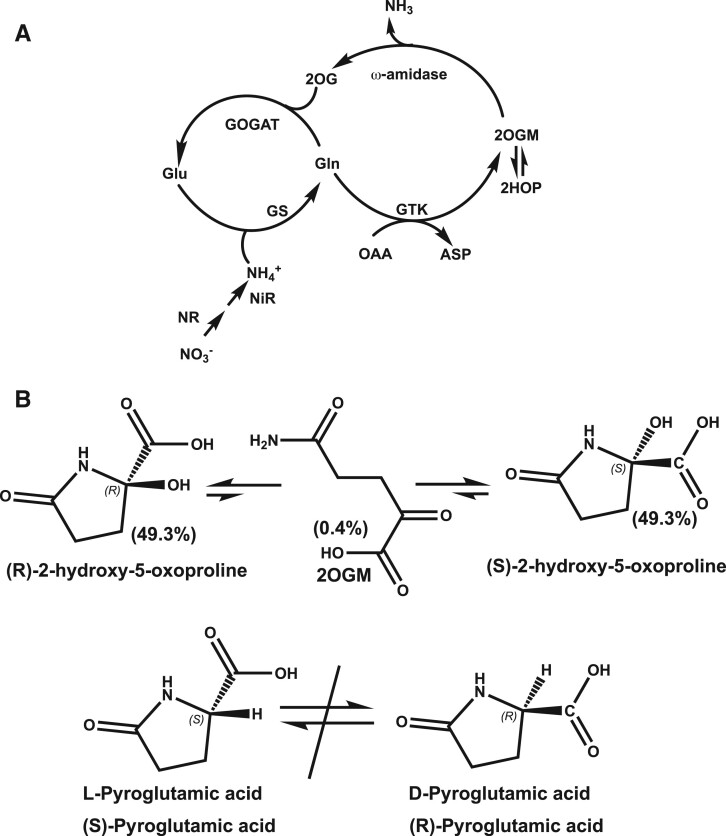
Ammonium assimilation and ω-amidase pathways and 2HOP and PGA structures. A, Ammonium assimilation and the ω-amidase pathways in plants. B, Equilibrium between 2HOP and 2OGM and the structures of a close structural analog, PGA. ASP, aspartic acid.

When nitrate availability to plants is increased, the expression of over 2,000 genes is changed (Canales et al., 2014). This coordinated cascade of gene expression changes includes induction of genes for increased nitrate uptake and assimilation, synthesis of key carbon skeletons associated with the tricarboxylic acid cycle (TCA cycle), and many other changes. These induced changes ultimately lead to increased photosynthetic capacity and biomass accumulation (Greenwood et al., 1986; Hirel and Krapp, 2020). This coordinated response is also consistent with plants actively assessing their internal N status (Gent and Forde, 2017). By sensing one or more N-metabolites, plants are likely to balance their C and N metabolisms and coordinate them with N uptake. All nitrate N moves into the plant’s metabolism through ammonium assimilation (Figure 1). Ammonium, generated by enzymatic reduction of nitrate and then nitrite, is assimilated by the action of glutamine synthetase (GS), which catalyzes the incorporation of ammonium into Glu to form Gln. Some of the Gln donates its amide N to 2-oxoglutarate (2OG) to form two molecules of Glu; this step is catalyzed by glutamate synthase (GOGAT).

The need for plants to actively assess their ammonium assimilation status has been appreciated for some time (Gutierrez et al., 2008; Vidal and Gutierrez, 2008; Gent and Forde, 2017). Here we investigated if a metabolite of Gln, 2-hydroxy-5-oxoproline (2HOP), acts as a signal of ammonium assimilation. 2HOP is generated by the Gln-dependent ω-amidase pathway (Figure 1) (Cooper and Meister, 1977). In the first step of the ω-amidase pathway, the amino N of Gln is transferred by Gln transaminase K (GTK) (EC 2.6.1.64) to a keto acid to form 2-oxoglutaramate (2OGM) and the corresponding amino acid. 2OGM is metabolized to 2OG and ammonium by ω-amidase (EC 3.5.1.3). 2OGM spontaneously cyclizes to form 2HOP (Cooper and Meister, 1977). The 2HOP ring opens to re-form 2OGM and thus the two molecules equilibrate (Hersh, 1971). 2HOP is thermodynamically favored; consequently, it accounts for most of the combined pools of 2OGM and 2HOP (Hersh, 1971; Cooper and Meister, 1977). For simplicity, we therefore refer here to the mixture of 2OGM and 2HOP as 2HOP. Nothing has been published about breakdown on 2HOP. The structure of the 2HOP molecule is unusual and distinctly different from any other metabolites of Gln or Glu (Figure 1B); this unique structure could provide the advantage of greater accuracy of recognition by a binding site.

Prior evidence of the ω-amidase pathway in plants includes reports related to GTK function. Crude plant extracts were found to synthesize 2OGM (Kretovich, 1958), whereas extracts of peas (*Pisum sativum*) were found to contain active GTK (Ireland and Joy, 1983). GTK protein was identified in the pea chloroplast proteome (Bayer et al., 2011). Recombinant Arabidopsis (*Arabidopsis thaliana*) GTK (At1g77670.1) was found to utilize 2-keto-4-methylthiobutyric acid to close the Yang cycle (Ellens et al., 2015). Omega amidase, the second enzyme in the ω-amidase pathway, was also found to be involved in the Yang cycle and in Asn and Ala metabolisms in plants (Zhang and Marsolais, 2014; Ellens et al., 2015). The Yang cycle was determined to be needed in reproductive tissues or when plants are stressed (Galston et al., 1996). Zhang and Marsolais (2014) found the Arabidopsis ω-amidase used 2OGM as a substrate. They also concluded this ω-amidase gene was widely expressed in Arabidopsis tissues.

Depending upon which of the two start codons in the Arabidopsis gene were used, GTK was found to be made with a plastid-targeting peptide that directed the GTK to the plastid or when the other start site was used, the GTK protein lacked the peptide and was found to be cytosolic (Ellens et al., 2015). Like GTK, Arabidopsis ω-amidase was identified to be localized in the cytosol and organelles because the ω-amidase gene also used alternate start codons (Ellens et al., 2015). The capacity to express GTK and ω-amidase in the cytosol and plastids is consistent with them being expressed where GS-catalyzed ammonium assimilation occurs. In the roots, ammonium assimilation is primarily catalyzed by GS1 in the cytosol; in the leaves, ammonium assimilation and re-assimilation are catalyzed by GS2 in the chloroplast (Bernard et al., 2008).

To determine if 2HOP, made by transamination of Gln, the first product of ammonium assimilation, is a potential metabolite signal of ammonium assimilation status, we examined the relationship between nitrate availability and the size of 2HOP pools in leaves and roots. We found that the 2HOP pool size in leaves increased as nitrate availability increased and that the size of the 2HOP pool in the roots was inversely related with nitrate availability; the importance of this is discussed. We also examined the effects of exogenously applied 2HOP. Ultimately, we constructed a model to explain how 2HOP affects plants.

## Results

### Foundational requirements for the intermediate in the ω-amidase pathway to be a signal

To determine if, as conjectured, 2HOP, the intermediate in the ω-amidase pathway, acts as a potential signal metabolite of the status of ammonium assimilation, the following four foundational requirements would have to be met:

- First, plants exogenously supplied with 2HOP would be expected to respond like plants with increased nitrogen availability.
- Second, the pool sizes of the pathway intermediate (2HOP) would respond to the availability of nitrogen.
- Third, the pool size of Gln, the 2HOP precursor, would reflect ammonium assimilation
- And fourth, the pathway and its intermediate would be broadly conserved among plants.

We designed the experimental investigation to test for these requirements and we report the results here.

### First: Plants responded to exogenously supplied 2HOP

**Figure 2:**
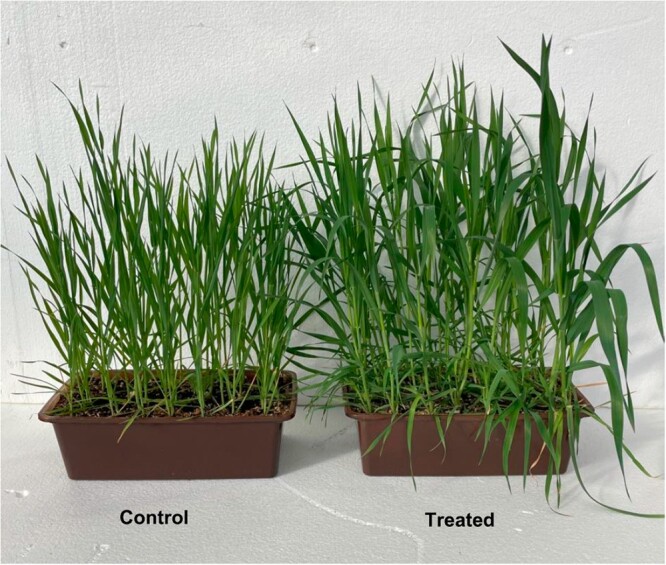
Control and 2HOP-treated oats. Each box contains 21 plants.

**Figure 3:**
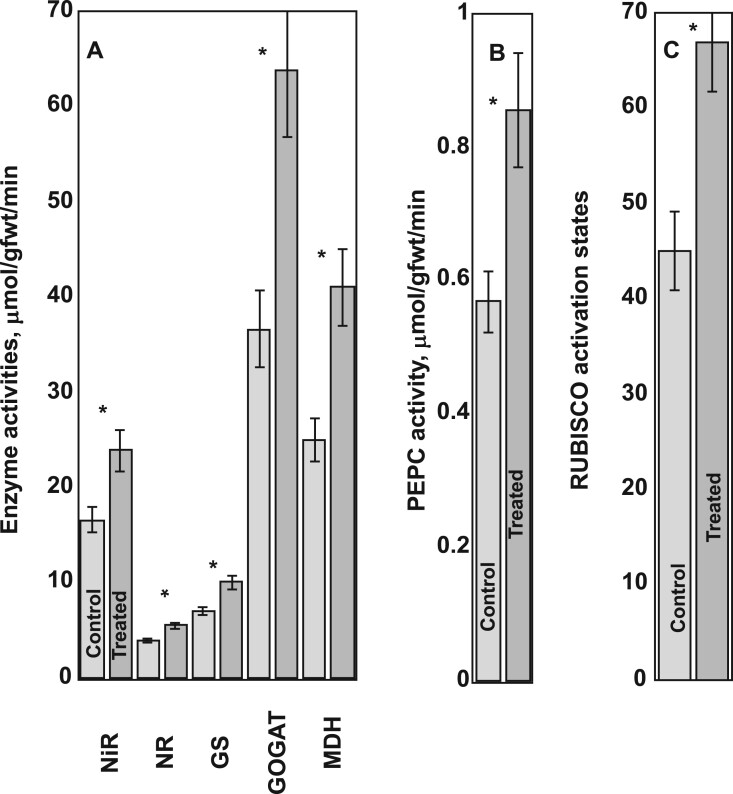
Effects of 2HOP on key enzyme activities in oat leaves. Twenty plants were used for each measurement. Student’s *t* test was used to determine the *P*-values that are provided in the figure for each comparison. A single asterisk represents *P ≤* 0.05. The error bars represent the standard deviation.

Greater plant biomass accumulated after treatment with 2HOP*.* Plants sprayed weekly with 100-μM 2HOP accumulated greater biomass than did untreated plants (Figure 2 and Table 1). The optimal concentration of 2HOP in the spray solution was identified (Supplemental Figure S1). In some early experiments daily sprays of solutions with 10-µM 2HOP were used (Figure 3); in later experiments weekly sprays with 100-µM 2HOP were used. These weekly sprays consistently generated good responses and provided experimental convenience. For these reasons, the weekly spraying method was adopted.

**Table 1:** Effects on key plant characteristics of treatment with 2HOP

Plants	Leaf 2HOP, nmol gfw^−1^, *P-*value	Root 2HOP, nmol gfw^−1^, *P-*value	Leaf Gln, µmol gfw^−1^, *P-*value	Root Gln, µmol gfw^−1^, *P-*value	Leaf nitrate µmol gfw^−1^, *P-*value	Leaf protein mg gfw^−1^, *P-*value	Chlorophyll µg gfw^−1^, *P-*value	CO_2_ fixation µmol m^−2^ s^−1^, *P-*value	Whole plant Fwt, g, *P*-value
Oats									
Control	1.225	0.680	0.615	0.582	115	6.0	996	13.5	8.9
Treated	1.669	0.394	0.827	0.791	59.0	8.8	1386	20.4	16.3
	0.026	0.029	0.32	0.037	0.014	0.026	0.038	0.021	0.023
Tobacco									
Control	3.348	1.573	8.534	3.615	69.5	4.6	831.6	8.9	19
Treated	4.506	0.498	13.166	5.906	22.8	6.9	1211	14.9	39.5
	0.025	0.007	0.028	0.021	0.007	0.036	0.033	0.019	0.006

The plants were greenhouse grown under natural light and provided with the complete nutrient solution described in the “Materials and methods” (10-mM nitrate). Sets of plants received weekly foliar sprays with or without 0.1-mM 2HOP beginning at the first true leaf stage. For whole plant fresh weight, 20 plants were sampled; for CO_2_ fixation and all other measurements, plants were individually sampled. The experiment was performed three times. Student’s *t*-test was used to determine the *P*-values.

Activities of enzymes of nitrate reduction and assimilation, CO_2_ acquisition, and enzymes associated with the TCA cycle increased after plant leaves were treated with 2HOP. As would be expected in plants that had taken up more nitrate, the activities of nitrate reductase (NR), nitrite reductase (NiR), GS, and GOGAT were increased in 2HOP-treated plants (Figure 3). These enzymes were recognized as a part of the primary nitrate response (Medici and Krouk, 2014). In the treated plants, the RUBISCO (ribulose bisphosphate carboxylase) activation state was increased. This increased activation state was consistent with the faster CO_2_ fixation rates (Figure 3 and Table 1). The activity of phosphoenol carboxylase (PEPC), another CO_2_-fixing enzyme that provides oxaloacetate (OAA) for the TCA cycle, was also increased as was the activity of an additional enzyme associated with the TCA cycle, malate dehydrogenase (MDH) (Figure 3).

Treatment with 2HOP stimulated changes in key physiological characteristics of plants with greater nitrate availability*.* These physiological changes are described in Table 1. The pools of Gln, the precursor of 2HOP, increased in the treated plant leaves and roots (Table 1). These increases were the same as in plants receiving additional N (Greenwood et al., 1986; Benin et al., 2012). The increased soluble leaf protein and chlorophyll contents (Table 1) were characteristic of plants receiving additional nitrogen (Lawlor, 2002). The treated plants had less nitrate stored in their leaves, which was consistent with their increased CO_2_ assimilation (Table 1) (Larios et al., 2004). The treated plants also accumulated greater biomass than untreated plants did (Table 1). These outcomes are characteristics of plants with greater nitrate availability. The plants assimilated greater amounts of nitrate as shown by their greater leaf protein (Table 1) and greater biomass. The treated plants contained elevated pools of 2HOP in their leaves. The estimated assimilated nitrogen per plant was calculated and is provided in the Supplemental Table S1. It was estimated that the untreated oats contained ∼7.09-g N per plant; in contrast, the treated plants contained ∼16.57-g N per plant. Additional ammonium assimilation data are provided in Supplemental Table S1.

The amount of 2HOP in the treatment solution (92 ng plant^−1^) was much less than the amount of leaf protein (94.2 mg plant^−1^) and therefore was not a nutritionally significant source of N. The treated plants were morphologically normal and showed no outward signs of any nutrient deficiencies.

Plants tested with other metabolites at the same concentration as 2HOP grew the same as untreated oats*.* When Glu, Gln, urea, Pro, or 2OG were substituted for 2HOP (100 µM) and sprayed weekly on young oats, the treated and untreated plants grew the same (Supplemental Figure S2). We did not apply high concentrations of Gln to the leaves and then measure 2HOP pools. Of course, if exogenous Gln were to enter the plant, it would be beneficial regardless of whether it was acted upon by GTK.

### Second: Leaf pools of 2HOP increased as nitrate availability increased; root pools of 2HOP were inversely related with nitrate availability

If 2OGM or 2HOP was a metabolite signal, its pool size could be expected to have a mutual relationship with the availability of nitrate and, consequently, with the ammonium assimilated into Gln given that Gln pools increase with greater nitrate availability (Greenwood et al., 1986; Benin et al., 2012). The 2HOP mixture was detectable by our method. The equilibrium between 2OGM and 2HOP heavily favors 2HOP as mentioned in the “Introduction” and described in Supplemental Figure S1.

**Figure 4:**
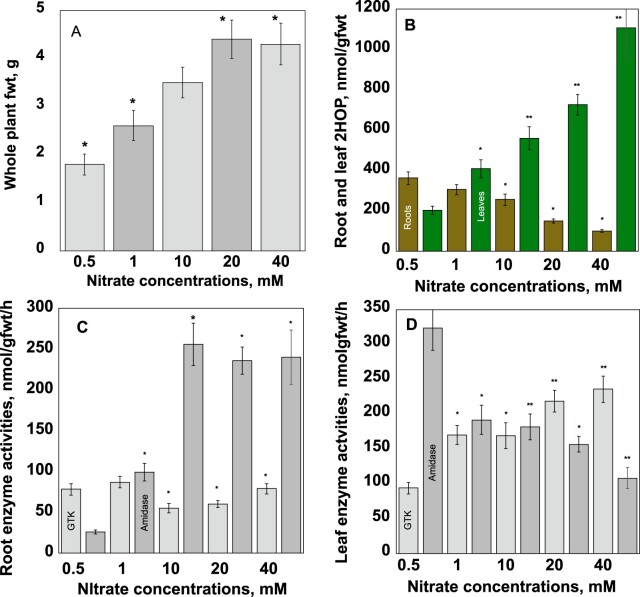
Responses to nutrient nitrate availability. In (A), the comparisons are with 10-mM nitrate; in (B–D) the comparisons are with the values at 0.5-mM nitrate. For whole plant fresh weights, 18 plants were used. For all other measurements, 15 plants were used for each measurement. The experiment was carried out three times. Student’s *t* test was used to analyze the data. A single asterisk represents ^*^P < 0.05 and a double asterisk represents ^**^P < 0.01. The error bars represent the standard deviation.

To test if the 2HOP pool sizes would respond to nitrate availability, oat seedlings were grown hydroponically and fed with different amounts of nitrate to create conditions in which increasing amounts of N were assimilated as approximated by increased biomass (Figure 4A). This approximation was ultimately supported by two key observations. The relationship between biomass, 2HOP levels, the pool sizes of the product of ammonium assimilation, Gln, and total ammonium assimilated was demonstrated (Table 1;Supplemental Table S2). The relationship between biomass and nitrate availability was also demonstrated (Figure 4).

**Table 2:** The ω-amidase pathway was active in leaves and roots of multiple plant species

Plant assayed	Leaf parameters			Root parameters		
	2HOP, nmol gfwt^−1^	GTK, nmol gfwt^−1^ h^−1^	ω-Amidase, nmol gfwt^−1^ h^−1^	2HOP, nmol gfwt^−1^	GTK, nmol gfwt^−1^ h^−1^	ω-Amidase, nmol gfwt^−1^ h^−1^
Oats	725 ± 106	219 ± 4	157 ± 12	149 ± 10	60 ± 4	236 ± 2
Tobacco	9,219 ± 794	259 ± 83	191 ± 0.9	5,514 ± 338	211 ± 35	252 ± 34
Maize (corn)	344 ± 48	149 ± 39	261 ± 18	101 ± 8	43 ± 7	157 ± 13
Arabidopsis	163 ± 26	132 ± 19	109 ± 11	NM	NM	NM
Tomato	105 ± 13	287 ± 31	NM	NM	NM	NM
Bean	NM	101 ± 11	NM	NM	NM	NM
Cowpea	NM	44 ± 21	NM	NM	NM	NM

Parameters are given as a function of fresh weight to allow comparison with 2HOP-treated plants, which contained elevated leaf protein content. The plants were grown in a greenhouse at 24°C under ambient light and provided with the complete nutrient solution described in the “Materials and methods” (10-mM nitrate). Oats, tobacco, and maize were more than 4 weeks old when sampled. The other plants were less than four weeks old. At least 12 individual plants were sampled for each measurement. NM, not measured. The symbol (±) provides the standard deviation.

The seedlings’ leaf 2HOP pools, GTK, and ω-amidase activities were measured (Figure 4, B–D). As predicted, the 2HOP pools in leaves increased as nitrate availability increased. At nitrate concentrations commonly found in soils (1 mM and 10 mM), the 2HOP synthesis and degradation enzyme activities were essentially equal (Figure 4) (Marschner, 1986). At higher levels of nitrate (20 mM and 40 mM), the GTK and ω-amidase activities in leaves changed to favor accumulation of 2HOP.

The seedlings’ root 2HOP pools, GTK, and ω-amidase activities were measured (Figure 4, B–D). In roots, the 2HOP pools were inversely associated with nitrate availability. The 2HOP pools in roots were highest at the lower (0.5 mM and 1 mM) and adequate (10 mM) nitrate concentrations; the 2HOP pools were lower at higher concentration (20 mM and 40 mM) of nitrate. The synthesis enzyme (GTK) activities were relatively low at lower nitrate availability and declined as nitrate availability increased. The 2HOP breakdown (ω-amidase) activity increased as nitrate availability was increased. The regulation of the GTK and ω-amidase activities was not determined.

### Third: The pool size of Gln, the 2HOP precursor, reflected ammonium assimilation

The source of the Gln for 2OGM and 2HOP synthesis in roots of C3 and C4 plants had to be primary ammonium assimilation because there was essentially no other substantial source; photorespiration does not function in the roots.

In C3 plant leaves, the Gln pool was a function of nitrate availability (Greenwood et al., 1986; Benin et al., 2012) even though Gln was synthesized during primary ammonium assimilation and during the re-assimilation step of photorespiration. This observation was consistent with the finding that changes in photorespiration did not impact the total Gln pool (Novitskaya et al., 2002). Thus, the Gln pool size could be expected to reflect the overall state of ammonium assimilation. The 2HOP pool increased as nitrate availability was increased (Figure 4).

**Figure 5:**
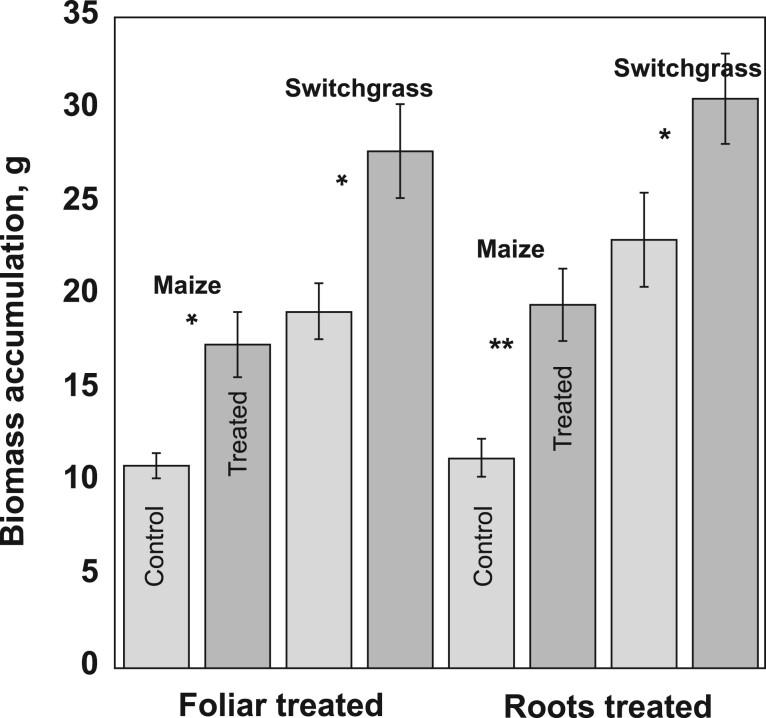
C-4 plants were treated with 2HOP had increased biomass. Twenty plants were used in each group. The experiment was carried out three times. Student’s *t* test was used to determine the *P*-values. A single asterisk represents ^*^P < 0.05; a double asterisk represents a ^**^P < 0.01. The error bars represent the standard deviation.

C4 plants carry out little photorespiration and thus ammonium assimilation would supply essentially all the Gln needed for 2HOP synthesis. Maize (*Zea mays*), a C4 plant, had 2HOP leaf and root pool sizes and GTK and ω-amidase activities comparable to those of the C3 plants examined, except for tobacco (*Nicotiana tabacum*) (Table 2). Highly active photorespiration was therefore seemingly not required for the ω-amidase pathway to function. Switchgrass (*Panicum virgatum*, another C4 plant) and maize also responded to treatment with 2HOP by accumulating greater biomass (Figure 5).

### Fourth: The ω-amidase pathway was broadly conserved among plants

To assess how well conserved the ω-amidase pathway was in plants and to obtain a basic biochemical characterization of the pathway, we tested several monocot and dicot plant species, including those with C3 and C4 metabolisms and legumes and nonlegumes. An active ω-amidase pathway was found in each of the plant species examined (Table 2). The GTK activity extracted from oat leaves used the following keto acids (in descending order): OAA (219 nmol gfwt^−1^ h^−1^, 100%), phenylpyruvate (69%), pyruvate (57%), and glyoxylate (46%). The standard assay for GTK activity was used and the different keto acids were substituted at the same molar concentration as OAA. The rate of production of 2HOP was measured for each keto acid and the rate was expressed as a percentage of the rate for OAA, the best keto acid.

The Arabidopsis GTK gene sequence at locus At1g77670.1 was overexpressed and the GTK enzyme was examined. In our experiments, the sequence beginning at the first start codon site was commercially codon optimized for expression in *Escherichia coli* and synthesized; we then overexpressed it using the pET-42a(+) vector in Agilent Technology’s Arctic Express (DE3) system. ^13^C NMR (carbon-13 nuclear magnetic resonance) was used to confirm that the accumulated product was 2HOP based on comparison with authentic 2HOP (synthesis described in “Materials and Methods”). Unfortunately, our experiments were conducted before Ellens et al. (2015) which showed that the first start site included a transit peptide directing the protein to plastids.

The recombinant Arabidopsis GTK enzyme activity was tested using different keto acids in the standard assay (see “Materials and methods”). The recombinant Arabidopsis GTK used the following keto acids in the same descending order as the oat GTK: oxaloacetate (2.8 nmol mg^−1^ h^−1^, 100%), phenylpyruvate (75%), pyruvate (55%), and glyoxylate (51%). This GTK had a relatively low Km for Gln (0.3 mM). No effort was made to fully purify the recombinant GTK.

Based on a BLASTp (basic local aleignment tool, protein sequence) search with the Arabidopsis GTK sequence, five additional GTK-encoding gene sequences were identified in genomes of soybean (*Glycine max*), rice (*Oryza sativa*), a moss (*Physcomitrium patens*), and *Chlamydomonas reinhardtii* and their function as GTK was confirmed (Supplemental Table S1). Each of these organisms contained active ω-amidase (Supplemental Table S1). Ellens et al. (2015) identified and confirmed the functions of GTK encoding genes in tomato (*Solanum lycopersicum*) and maize.

GTK should not be confused with the Asn aminotransferase activity of ATG1 (alanine: glyoxylate aminotransferase), also known as serine-glyoxylate aminotransferase (*A. thaliana*, Accession No. AF06390) (Zhang et al., 2014). Indeed, a pairwise sequence comparison (EMBOSS-Water) of the protein sequences of ATG1 (Liepman and Olsen, 2001) and GTK (*A. thaliana*, At1g77670.1) revealed only 20.3% identity, 33.4% similarity, and 38.2% gaps.

A sequence (At5g12040) had been identified as an ω-amidase in the KEGG database and Zhang and Marsolais (2014) confirmed that this sequence encodes ω-amidase and uses 2OGM as a substrate. With this sequence as the basis, a BLASTp search identified possible ω-amidase sequences with high percentage identities and positives in soybeans, rice, a moss, and *C. reinhardti* (Supplemental Table S1). Ellens et al. (2015) identified and confirmed the functions of ω-amidase encoding genes in tomato and maize.

### Nitrate uptake rate increased after treating roots with 2HOP

**Figure 6:**
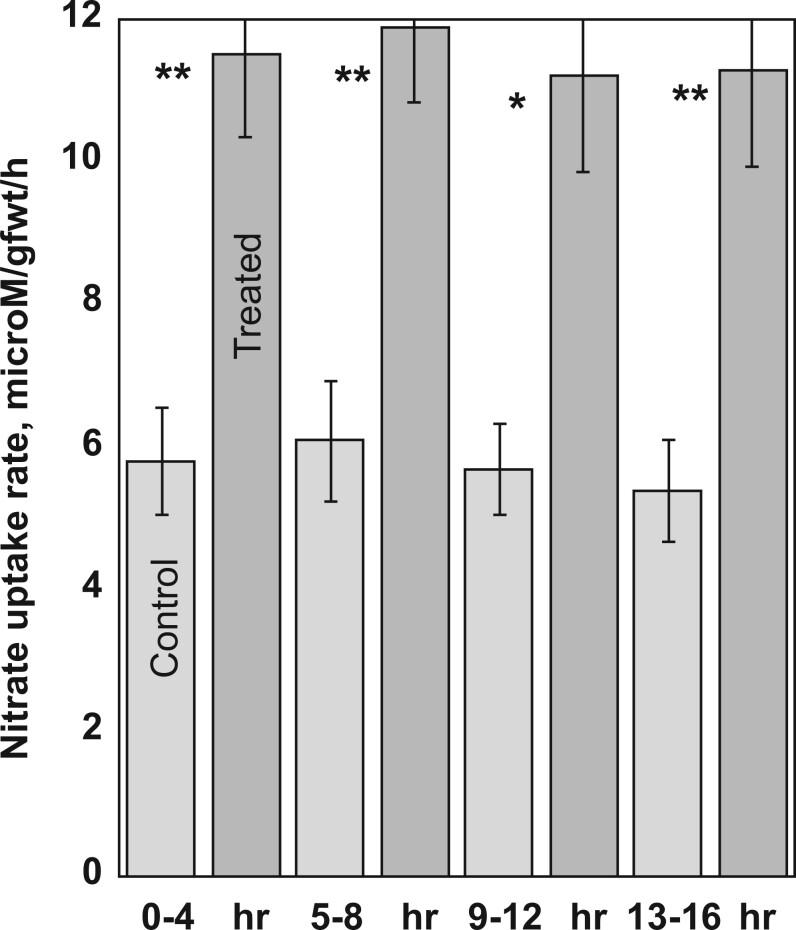
Stimulation of nitrate uptake by providing 2HOP to oat roots. The experiment was repeated four times; 16 plants were used for each measurement. Student’s *t* test was used to determine the *P*-values which are represented as a single asterisk for ^*^P < 0.05 and a double asterisk for ^**^P < 0.01. The error bars represent the standard deviation.

The effect of exogenous 2HOP provided to the roots on the nitrate uptake rate was examined in oat seedlings grown hydroponically (Figure 6). The treated seedlings took up nitrate at essentially twice the rate of untreated plants in each of the four 4-h measurement periods. Nitrate uptake increased quickly after the treatment began, as shown by the similar rates in each of the four measurement periods. If the increase in the uptake rate had occurred slowly, the rate in the first period would be expected to be slower than the rates in the subsequent periods. The greater assimilated N, as shown by greater biomass and higher leaf protein content (Table 1; Supplemental Table S2) was consistent with foliar-applied 2HOP ultimately stimulating greater overall nitrate uptake and assimilation.

### R-2HOP and S-2HOP are likely to be active in the plant because their structural analogs are active

In solution 2HOP was in equilibrium with the open-chain molecule, 2OGM (Figure 1B) (Hersh, 1971). Consequently, it was unclear which molecule or molecules were the active. Some clarity was obtained by testing a close structural analog of 2HOP. Pyroglutamic acid (PGA) differs from 2HOP only at C2 where it has a hydrogen in place of the OH. PGA is present as L and D isomers; each of these isomers can be isolated and used separately. The L-PGA and D-PGA which were analogous to R-2HOP and S-2HOP, respectively (Figure 1B), were each active in driving faster leaf emergence rates. We conclude from these results that R-2HOP and S-2HOP are likely to be active in the plants. Greenhouse-grown oat seedlings were either not treated and served as controls or were sprayed weekly with either 100-µM L-PGA or 100-µM D-PGA and the leaf emergence rates were compared with those of untreated plants. Student’s *t* test was used to examine the data. The untreated plants had leaf emergence rates of 0.290 leaves day^−1^ plant^−1^. The p values versus treatments were <0.05. The plants treated with L-PGA had leaf emergence rates of 0.343 leaves d^−1^ plant^−1^ (the *P*-value versus D-PGA was <0.05). Those treated with D-PGA had leaf emergence rates of 0.321 leaves d^−1^ plant^−1^. Twenty plants were used in each set; the experiment was repeated three times.

### Effects of 2HOP foliar sprays were sustained for at least 3 weeks

**Figure 7:**
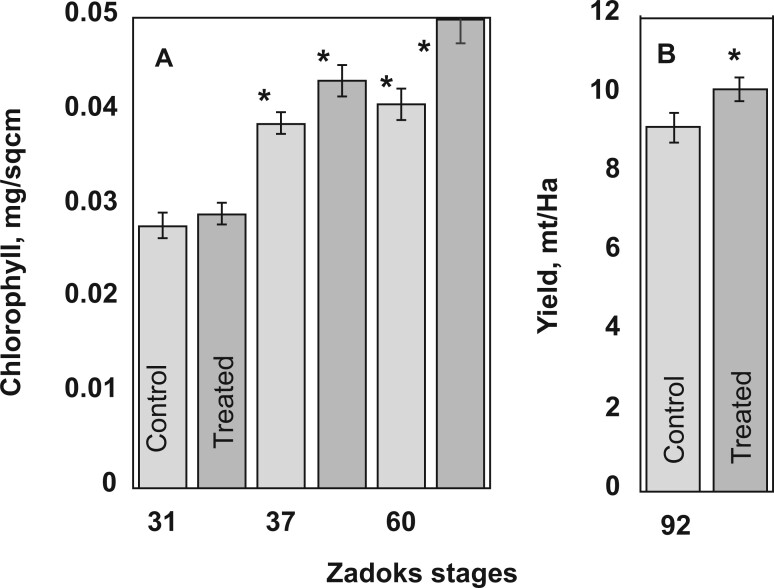
Field trial with winter wheat shows the prolonged effects of a single foliar treatment with a mixture of 2HOP and L-PGA. The mixture was applied at Zadocks. Thirty-one and therefore in (A) comparisons are made with values at this developmental stage. In (B) the comparison if between control and treated. Duncan–Waller *t* test was applied to determine the *P*-values. A single asterisk represents ^*^P < 0.05. The error bars represent the standard deviation.

The previously described experiments were conducted in the greenhouse with weekly treatments of 2HOP. A field trial was used to test if a single treatment of 2HOP plus PGA would drive sustained changes in the plants and to determine if a single treatment during vegetative growth would initiate changes of sufficient magnitude to increase seed yield. This field trial was conducted because field trials are more reliable than greenhouse tests in determining grain yield. 2HOP plus L-PGA was sprayed on field-grown wheat (*Triticum aestivum*) at Zadoks growth stage 30/31 when stems were elongating, and nodes were developing. Twenty-one days after the single treatment, at Zadoks 60, the treated plants had greater chlorophyll content (Figure 7). They also produced a higher yield of grain, which is like crops fertilized with a greater amount of N fertilizer (Benin et al., 2012).

## Discussion

The substantive result of this experimental study was that plants supplied adequate nitrate and provided with micromolar solutions of 2HOP grew more than did untreated controls (Figure 2 and Table 1). All the results presented here are consistent with 2HOP having two functions in plants: one as a metabolite signal for ammonium assimilation status and the other as a nitrate uptake stimulant.

**Figure 8:**
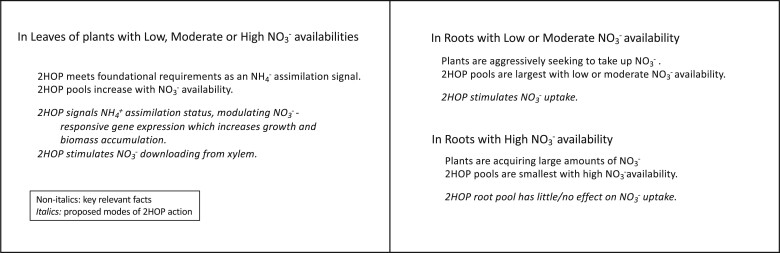
Proposed modes of 2HOP actions 2HOP met the foundational requirements of an ammonium assimilation signal only in leaves; therefore, 2HOP acting as a signal is proposed only in the leaves. 2HOP stimulation of ${\text{NO}}_{3}^{-}$ uptake by roots was proposed based on our finding that exogenous 2HOP stimulated ${\text{NO}}_{3}^{-}$ uptake by roots. 2HOP stimulating ${\text{NO}}_{3}^{-}$ downloading from the xylem was proposed because this process and nitrate uptake by roots each utilize nitrate transporters. Therefore, the nitrate transporters in the leaves, like those in roots, could be stimulated by 2HOP. Nitrate availabilities: low is 0.5 mM and 1 mM; moderate is 10 mM; high is 20 mM and 40 mM nitrate in the nutrient solutions.

Model of 2HOP actions and their consequences. Our results discussed below allowed us to construct a basic model of how 2HOP may be acting in leaves and roots (Figure 8). There are two key findings in support of the model. First, 2HOP met the four foundational requirements for it to be a signal metabolite of ammonium assimilation. Second, 2HOP stimulated nitrate uptake into roots treated with 2HOP. Data supporting the model are presented in Figure 8.

2HOP as a possible signal of ammonium assimilation status. Four foundational requirements were defined for 2HOP, a metabolite of Gln, to possibly be a signal of the status of ammonium assimilation. These requirements were met in leaves. First, plants provided 2HOP by foliar spray, responded like plants with increased nitrate availability by increasing key enzyme activities in their N and C metabolisms and CO_2_ fixation (Figures 3 and 4) and ultimately, by accumulating greater biomass (Figures 2 and 5). Second, the 2HOP pool in leaves responded positively to nitrate availability (Figure 5). Third, the pool size of Gln, the precursor of 2HOP, reflected ammonium assimilation. Fourth, we observed strong conservation of the ω-amidase pathway in a broad range of plants (Table 2). A metabolite signal of ammonium assimilation status in cereal leaves could be useful because cereals reduce and assimilate approximately two-thirds of their nitrate in their leaves and the remaining one-third in their roots (Gojon et al., 1986; Scheurwater et al., 2002; Andrews et al., 1992).

We have not identified the mechanism(s) by which 2HOP could act as a signal of ammonium assimilation status, but there are several possibilities. 2HOP could interact with one or more transcription factors or with Glu receptors that may be involved in sensing N status (Gent and Forde, 2017).

2HOP as a nitrate uptake stimulant. Exogenous 2HOP stimulated nitrate uptake into roots (Figure 4). Larger 2HOP pools in roots were found when nitrate availability was low (1 mM) to adequate (10 mM), suggesting that 2HOP could stimulate nitrate uptake at these availabilities. As a matter of practicality for the plant, these are the same levels of nitrate availability found in agricultural and unmanaged soils (Marschner, 1986). If 2HOP stimulates nitrate uptake under these conditions, the increased nitrate taken up would be expected to increase the expression of the nitrate-responsive genes, which would be consistent with our observation of increased activities of several enzymes whose genes are nitrate responsive after treatment with 2HOP (Figure 4).

In leaves, 2HOP pools were larger either when nitrate availability increased (Figure 5) or when plants were treated with 2HOP (Table 1). It is possible that these larger 2HOP pools stimulated nitrate downloading from the xylem; this is analogous to 2HOP stimulating nitrate uptake in roots. If true, this greater influx of nitrate would be expected to increase the expression of the nitrate-induced genes. When plants were given foliar applications of 2HOP they also increased their overall nitrate uptake as shown by their greater leaf protein content/gfwt and greater biomass (Table 1;Supplemental Table S2). The total assimilated N in untreated oats was 7.9 ± 0.8 g plant^−1^; in treated oats, it was 16.57 ± 1.3 g plant^−1^ (Supplemental Table S2). How foliar-applied 2HOP ultimately stimulated greater nitrate uptake was not investigated. It is possible that 2HOP was translocated to the roots or it may have interacted with other shoot-to-root signaling mechanisms such as the cytokinins (Vidal et al., 2020).

It is possible that 2HOP acts post-translationally to stimulate nitrate uptake. Supporting this possibility is our finding that the nitrate uptake rate increased quickly as shown by the first 4-h rate being the same as the next three 4-h rates. Also, the plants were growing with adequate nitrate that itself would already have induced nitrate transporter gene expression (Wang et al., 2003). On one hand, 2HOP could impact the phosphorylation of the nitrate transporter, NRT1.1, or other nitrate transporters, which converts them from low- to high-affinity transporters (Jacquot et al., 2017). On the other hand, 2HOP may be an allosteric effector of the nitrate transporter accessory protein (NAR), NAR2 (Jacquot et al., 2017). Either way, by stimulating nitrate uptake, 2HOP, a product of ammonium assimilation can drive the entire nitrate response.

## Conclusion

In this study, we employed a multidisciplinary approach to investigate possible functions of HOP, a metabolite made directly from Gln, the first product of ammonium assimilation in plants. The results of this investigation were consistent with 2HOP having two possible functions in plants: as a signal of the status of ammonium assimilation and as a nitrate uptake/transport stimulant (Figure 8). First, 2HOP met the foundational requirements of a signal molecule that reflects the status of ammonium assimilation in leaves; 2HOP signaling the status of ammonium assimilation in leaves was therefore proposed. Second, because exogenous 2HOP stimulated nitrate uptake and because the root 2HOP pools were largest when nitrate was at low or moderate availability, 2HOP stimulating nitrate uptake/transport into roots was proposed. 2HOP stimulating ${\text{NO}}_{3}^{-}$ downloading from the xylem was proposed because this process and nitrate uptake by roots each utilize nitrate transporters. Nitrate transporters in the leaves, like those in roots, could therefore be stimulated by 2HOP.

Given the deepening climate change crisis, global agriculture, like all other energy-intensive human activities, must reduce its overall carbon footprint. A major contributor to agriculture’s massive carbon footprint is its heavy reliance on large amounts of nitrogen fertilizers to increase yield. Production of nitrate and ammonium fertilizers is extremely energy intensive and much of that energy is produced from burning carbon-based fuels. 2HOP could potentially contribute to climate change solutions because 2HOP-treated plants out-yield untreated plants. The carbon footprint per unit of yield of 2HOP-treated plants would be lower. Moreover, 2HOP-treated plants were found to be aggressive at taking up nitrate, which would potentially reduce the use of nitrate fertilizer per unit of yield, leading to a further reduction in agriculture’s carbon footprint.

## Materials and methods

### Recombinant GTK and ω-amidase productiond

The GTK sequence (At1g77670.1, BT028918.1) was used for a BLAST-X analysis to find sequences in other plants and organisms with high homology. The Arabidopsis ω-amidase (At5g12040, AY093711.1) was used for a BLAST-X analysis for sequences in other plants and organisms with high homology.

The GTK or ω-amidase gene sequences were codon optimized and synthesized commercially and expressed using the pET-42a (+) vector in the Arctic Express (DE3) system (*E. coli*) (Agilent Technologies). After induction with IPTG (isopropyl β-D-1-thiogalactopyranoside) (1 mM), the cells were grown for 24 h at 10°C and collected with centrifugation (6,000*g*, 10 min), washed with PBS (phosphate buffered saline), centrifuged again, and resuspended in 150-mM Tris–HCl, pH 8.5 containing 1-mM β-mercaptoethanol and 200-µM pyridoxal phosphate and lysed with sonication (3 × 30 s). Cell debris were removed by centrifugation (12,000*g*, 10 min). The supernatant was stored at −80°C.

### Confirmation of recombinant Arabidopsis GTK function

We confirmed that the GTK protein resulting from the Arabidopsis (*A. thaliana*) *gtk* sequence (At1g77670.1) produced 2HOP as determined by ^13^C NMR. The reaction mixture contained 150-mM Tris–HCl, pH 8.5, 1-mM β-mercaptoethanol, 200-mM Gln, 100-mM glyoxylate, and 200-µM pyridoxal 5′-phosphate. The higher concentrations were used to ensure that the product would be sufficiently concentrated to be detectable by ^13^C NMR. The GTK and no-GTK control reaction mixtures were incubated at 37°C for 20 h and clarified by centrifugation (10,000*g*). Supernatants were examined for the production of 2HOP; authentic chemically synthesized 2HOP was used as a reference. We observed the disappearance of resonances attributable to Gln and glyoxylate and the appearance of resonances characteristic of 2HOP. Particularly diagnostic of 2HOP was the acetal carbon (Cα) at 86.9–87.0 ppm (see synthesis below). The pH stability of 2HOP was established.

The keto acid preferences for the Arabidopsis GTK were determined using the assay described below. The GTK enzymes resulting from the sequences from barley (*Hordeum vulgare*), soybean (*G. max*) and rice (*O. sativa*) were assayed using our assay described below.

### Assays of enzyme activities

Enzyme activities from plants were made using crude extracts. All assays were linear during the period examined and all were done in triplicate for each sample from each plant. For GTK activity, leaf and root samples (w:v of 1:3) were ground in extraction buffer consisting of 25-mM Tris–HCl, pH 8.5, 1-mM EDTA (ethylenediaminetetraacetic acid), 20-µM FAD, 10-mM cysteine, and 1.5% (v/v) mercaptoethanol. After centrifugation at 10,000*g*, the sample was added to the assay mixture (1 mL total) containing 20-mM Gln, 0.4-mM OAA, 0.2 pyridoxal phosphate, 150-mM sodium borate buffer (pH 8.5), and incubated for 30 min at 37°C (Calderon et al., 1985).

HPLC (high-performance liquid chromatography) was used to quantify the 2HOP using an ICSep ICE-ION-300 7.8-mm ID × 30-cm L column (TRANSGENOMICS) and eluted with 0.01 N H_2_SO_4_ at a flow rate of 0.2 mL min^−1^ at 40°C. The injection volume was 20 µL; the retention time was between 38 min and 39 min. Detection was achieved with 210-nm light. Control assays with either pyridoxal phosphate or Gln omitted were used and neither produced 2HOP. Authentic 2HOP and the product had the same chromatographic retention times. 2HOP and the amino acid corresponding to the keto acid used were generated.

ω-amidase activity was extracted from a sample (w:v of 1:3) ground in extraction buffer containing 0.1 mM EDTA, 5-mM 2-mercaptoethanol, and 50-mM Tris–HCl, pH 8.5). After centrifugation at 10,000*g*, the sample was added to the assay mixture (1 mL) consisting of 10-mM 2OMG, 2-mM mercaptoethanol, 50-mM Tris–HCl, pH 8.5), and incubated for 30 min at 37°C; the reaction was stopped with 0.1 mL of 17% (v/v) TCA (Calderon et al., 1985). The disappearance of 2HOP and the appearance of 2OG were monitored using the HPLC method described above for the GTP assay.

Enzyme activities were measured according to published procedures for GS (Shapiro and Stadtmann, 1970), NR (Long and Oaks, 1990), NiR (Wray and Filner, 1970), PEPC, MDH, and ME (malic enzyme) (Merlo et al., 1993), and the RUBISCO activation state (Sharkey et al., 1991). Enzyme activities were measured using a minimum of three plants. The fourth or fifth leaf counting from the apical cluster was used in tobacco. For other plants, mature, fully expanded leaves were used.

### Synthesis of 2-hydroxy-5-oxopyrrolidine-2-carboxylic acid (2HOP) and 2OGM

2OGM and 2HOP were synthesized in a single step by the action of Fremy’s salt (commercially available potassium nitrosodisulphonate) on PGA. A buffer solution was prepared with 1.3-M NaHCO_3_ to which 10-M NaOH was added to set the pH to approximately 9.5. Next, 30.0 g of PGA was dissolved, with moderate stirring, in 500 mL of the sodium bicarbonate buffer in a 2-L round-bottom flask. Approximately half of the Fremy’s salt (62.34 g) and an additional 300 mL of the sodium bicarbonate buffer were added. The pH of the solution was maintained at between 9.0 and 9.5. The reaction was stirred at room temperature for 30–48 h or until the solution became colorless, at which point the other half of Fremy’s salt was added and the reaction was allowed to continue for another 30–48 h or until the solution became colorless again. ^13^C NMR spectra were obtained to follow the progress of the reaction. Additional Fremy’s salt could be added, if necessary, to drive the reaction to completion. The proton form of Dowex 50 × 8 resin was added with stirring, until the pH was 4.5. The resin was removed by filtration; the supernatant was dried; and the 2HOP was isolated by extraction with dimethylsulfoxide. The synthesis yielded approximately 30 g of 2HOP.

### ^13^C NMR characterization of 2HOP

Chemical shift assignments were made using a ^13^C DQF COSY (Double quantum filtered correlated spectroscopy) spectrum of 2-[U-^13^C_5_] hydroxy-5-oxoproline; they were as follows: C_1_ 175.3 ppm; C_α_ 87.0 ppm; C_β_ 31.1 ppm; C_γ_ 27.5 ppm; C_δ_ 179.4 ppm. The carbons are labeled in the 2HOP structure provided in Supplemental Figure S3. Spectra were obtained at pH values from 5.0 to 11.0; only the closed-ring form (2HOP) was evident in the spectra.

### Plant growth and treatment with 2HOP and PGA

Oat *(Avena sativa*) variety Lodi was used wherever oats were studied including Figure 2.

#### Nutrient media

The nutrient media described here was provided to all of plants expect for experiments in Figures 4 and 6 when modifications are described. It contains 10-mM nitrate as the sole N source. It was otherwise comparable with Hoagland’s media. It contained the macronutrients calcium chloride (4 mM), monobasic potassium phosphate (2.5 mM), magnesium chloride (0.2 mM), potassium sulfate (4 mM), and potassium nitrate (10 mM) and the micronutrients ferrous chloride (0.065 mM), manganese sulfate (0.006 mM), boric acid (0.03 mM), zinc sulfate (0.0015 mM), copper sulfate (0.0002 mM), and molybdenum oxide (0.0002 mM).

#### 2HOP treatments

For the experiment reported in Figure 3, daily (14 days) foliar sprays containing 10−µM 2HOP were applied to the seedlings. For all other greenhouse-grown plants, the plants were treated weekly with foliar applications of 2HOP (100 µM) solution, pH 6.5–7.5 containing (0.1% w/v) sodium docecyl sulfate and (1.25% v/v) glycerol. The control plants were sprayed with SDS and glycerol without the 2HOP. The solution was sprayed until dripping from the foliage. For the field trial reported in Figure 7, the plants were sprayed with an application rate of 100 g ha^−1^.

In Table 1, the plants were grown in a growth chamber (24C) with 16-h light and 8-h darkness. Oats were treated weekly, beginning at first true leaf emergence and the treatment continued for 28 days. Tobacco seeds were germinated in phytotrays containing Murashige and Skoog medium and grown in sand culture. Tobacco treatment began after adaptation to ambient conditions.

In Table 2, the plants were grown in a greenhouse maintained at 24°C with ambient light. They were fed the same nutrient solution described in detail for Figure 3.

In Figure 2, the plants were grown in a greenhouse under ambient light. They were provided our standard nutrient solution. Four weekly sprays with 100-mM 2HOP were begun at the four-leaf stage.

In Figure 3, the plants used for key enzyme activities were grown in a growth chamber (24°C) (16-h light and 8-h dark) under conditions described previously for oats (Knight et al., 1988) and tobacco (Temple et al., 1993). Daily (14 days) foliar sprays containing 10−µM 2HOP were applied to the seedlings.

The experiments in Figure 4 examined the effects of a range of concentrations of nitrate. The experiment required nutrient solutions containing 0.5-, 1-, 10-, 20-, and 40-mM KNO_3_. The nutrient solution described above was modified as follows to prepare the required nitrate concentrations. For lower concentrations of nitrate, the concentration of potassium sulfate was increased to provide the potassium no longer provided by potassium nitrate. For the higher concentrations of nitrate, only potassium nitrate was increased. Oat seedlings were gown hydroponically in these media in a greenhouse under ambient light.

In Figure 5, the plants used to test the effects of 2HOP on plants with C4 metabolism were Switchgrass (*P. virgatum* var. Alamo) and maize (*Z. mays* var. Northstine Dent). These plants were grown in a greenhouse at 24°C with natural light and provided the nutrient solution described above in the growth chamber experiment. 2HOP (100 µM) was applied as weekly foliar sprays. For the root system treatments, 750 nmol of 2OP was provided daily. Twenty plants were used in each set within each experiment. The foliar treatment contained 100-µM 2HOP applied weekly. For the root treatment, 750 nmol of 2HOP was provided daily.

See the section below “Measurement of nitrate uptake rate” for Figure 6.

In Figure 7, the effects of 2HOP plus PGA were tested in a field trial during 2019–2020. The chemicals were applied to foliage at a total of 100 g ha^−1^. Winter wheat (*T. aestivum*) variety KWS Saki was grown in three replicated 20 m^2^ plots with appropriate border zones in the Suffolk region of the UK. Fertilization was according to the farmer standard with all plots receiving the same amount.

### Measurement of nitrate uptake rate

To measure nitrate uptake rates (Figure 6), the plants were grown by the method of Ward et al. (1986). Their method as we implemented it, is described here. Oats seedlings were grown hydroponically and provided the complete nutrient solution described in “Materials and methods”. The oat seedlings were shifted to no N nutrient solution while they were shifted to continuous darkness for 7 days followed by 2 days of continuous light to minimize any effect of circadian rhythms. In the no N nutrient solution, potassium nitrate was replaced with potassium sulfate on an equimolar basis. Continuous light was used for the remainder of the experiment.

Four groups (one for each time point) of 12 seedlings of uniform size were transferred to nutrient solution with 1-mM KNO_3_ for 12 h and then transferred to the nutrient solution with 0.75-mM KNO_3_ and without 2HOP (24 mL). An additional four groups (one for each time point) of 12 seedlings were transferred to the nutrient solution with 1-mM KNO_3_ for 12 h and then transferred to the nutrient solution with 0.75-mM KNO_3_ and with 1-mM 2HOP (24 mL). After each 4-h period, one group of seedlings from untreated and one group from 2HOP-treated seedlings were harvested and their biomass determined and the loss of nitrate from their respective nutrient solution was measured.

The disappearance of nitrate from the solutions was detected and quantified by HPLC with ultraviolet detection (Thayer and Huffaker, 1980). The HPLC was fitted with a strong anionic exchange (Partisil-10 SAX column 25 cm × 4.6 mM id, Sigma Millipore). The isocratic separation used 50 mM phosphate buffer, pH 3.0 that had been filtered through a 0.45-micron membrane filter as the eluent. The flow rate was 1 mL min−. Samples of 50 µL were injected using an injection loop. The nitrate was detected at 210 nm, taking advantage of the high ultraviolet absorbance of nitrate at 210 nm.

The sensitivity of the method was 1.25 µM in the target solution (Thayer and Huffaker, 1980). Thus, the method is more than adequate to detect the changes we observed.

The entire experiment was done four times each on consecutive days.

### Assays of chlorophyll, nitrate content in leaves and roots and protein

Chlorophyll content was measured as described by Arnon (1947). The nitrate pool size was measured 6 h after the beginning of the light period (Cataldo et al., 1975). Protein was measured by the method of Bradford (1972).

### Extraction and assay of 2HOP from fresh leaves and roots

The 2HOP was readily extracted from fresh plant samples with acidified water (H_2_SO_4_). The samples were ground with a mortar and pestle with water (1:2 wt:vol). The ground material was then centrifuged (10,000*g*, 10 min). Each sample was passed through a 0.45-micron filter. HPLC was used to detect and quantify it as described in the GTK activity assay. Authentic 2HOP was added to a control extract to show it would be recovered and detected. PGA was also added to a control extract. 2HOP and PGA are very water soluble; each could be expected to be extracted if present in the samples. We have been careful to refer to using 2HOP, the equilibrium mixture, and interpreting the results as being attributable to 2HOP, the equilibrium mixture.

### Measurement of CO_2_ fixation

CO_2_ fixation rates were measured 4–7 h after the beginning of the light period using a PPSystems CIRAS closed photosynthesis system at 2,000 microE light intensity for 20 min. Measurements were made using at least four plants. The measurements were made on fully opened leaves in the same position on the plant relative to the top of the plant.

### Sampling and population sizes

These are given for each experiment in the legend of the figure or table reporting the data.

### Accession numbers

Sequence data from this article can be found in the GenBank/EMBL data libraries under accession numbers that are provided in Supplemental Table S2.

## Supplemental data

The following materials are available in the online version of this article.

**Supplemental Figure S1**. Identifying the 2HOP treatment concentrations to which plants respond.

**Supplemental Figure S2**. Comparison of the effects of Glu, Gln, Pro, 2OG, and 2HOP application (100 mM) on leaf emergence rates.

**Supplemental Figure S3**. Structure of 2HOP with the carbons labeled.

**Supplemental Table S1**. GTP and *ω* -amidase homologues identified in plants, a moss, and an alga.

**Supplemental Table S2**. Estimation of assimilated N in untreated and treated plants.

## Supplementary Material

kiac501_Supplementary_Data

## Notes

P.J.U. theorized the pathway function in plants and the effect of the intermediate, designed, and conducted experiments, interpreted results, and wrote the paper. T.J.K. designed and conducted experiments and interpreted results. R.A.M. conceived and carried out the chemical synthesis of the pathway intermediate and critically read the manuscript.

The author responsible for distribution of materials integral to the findings presented in this article in accordance with the policy described in the Instructions for Authors (https://academic.oup.com/plphys/pages/general-instructions) is Pat J. Unkefer (pjunkefer@gmail.com).
