# Supplementary material for: The intermediate in a nitrate-responsive ω-amidase pathway in plants may signal ammonium assimilation status
Source: Plant Physiol. 2022 Oct 27;191(1):715–28. doi: 10.1093/plphys/kiac501 (PMC9806585; doi:10.1093/plphys/kiac501)
Supplement: kiac501_Supplementary_Data [file kiac501_supplementary_data.pdf]

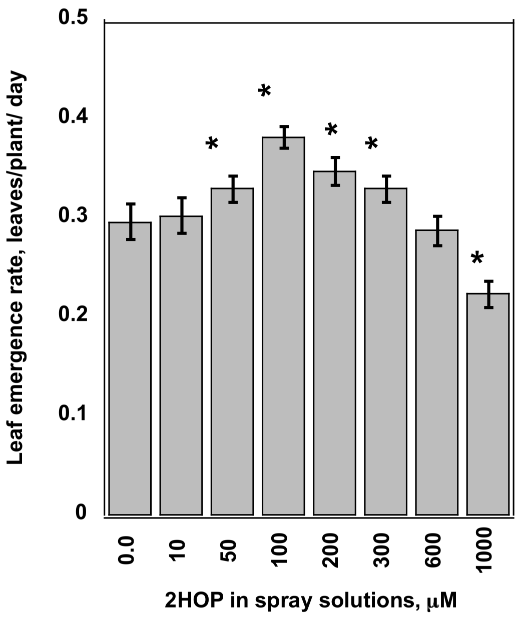

Supplemental Figure S1. *Identifying the 2HOP treatment concentrations to which plants responded.* The plant growth rate was assessed by tracking the leaf emergence rate. Oat seedlings were grown in a greenhouse under ambient light at 24C. All plants were provided a complete nutrient solution that contained 10 mM nitrate. 2HOP was applied via a weekly foliar spray beginning at the 2-3 leaf stage. Plants were allowed to grow for 25 days after the first treatment.

30 plants were examined at each concentration of 2HOP. The experiment was repeated three times. The error bars represent the standard deviation Student's t-test was used to determine the p values which are represented as a single asterisk (*) for $p<0.05$. The error bars represent the standard deviation.

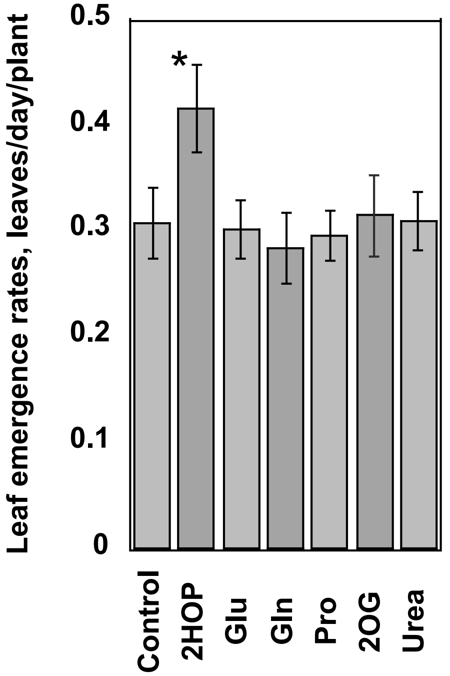

Supplemental Figure S2. *Comparison of the effects of Glu, Gln, Pro, 2-oxoglutarate (2OG) and 2HOP applied (100μM) on leaf emergence rates.* Glu, Gln, and 2OG were tested because of their involvement in ammonium assimilation. Pro was tested because of its structural similarity with 2HOP. Urea was tested because it is a good nitrogen source. Of course, providing Gln or most amino acids in much higher concentrations is beneficial to plants.

Oats were grown in a greenhouse at 24 C under ambient light and provided a complete nutrient solution containing 10 mM nitrate. Plants were given 2 weekly foliar sprays beginning at 2 leaf stage. Each compound was applied at 100 μM concentration. Leaf emergence was tracked beginning the day of the first spray and continued for 21 days. 20 plants were used for each compound or control. The experiment was repeated three times. Student's t-test was used to determine the p values which are represented as a single asterisk (*) for $p<0.05$. The error bars represent the standard deviation.

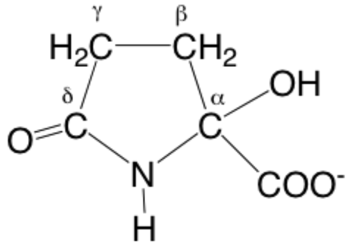

Supplemental Figure S3. *The structure of 2-hydroxy-5-oxoproline (2-HOP) with the carbons labeled to correspond with interpretation of the ^{13}CNMR spectra.*

Supplemental Table S1. GTP and ω-amidase homologues Identified in plants, a moss and an alga

[a]Gene sequences were expressed in *E. coli using the Arctic Express system* and their activities were assayed. Wildtype *E. coli* control GTK activity was 0.02 nmol/mg/h. No effort was expended to fully recover the activity as confirmation of the function of the expressed protein was the goal.
[b]Wildtype plant has measured GTK activity (ω-Amidase Pathway).

Species	NCBI Accession Number		GTK			ω-amidase	
	GTK	ω-amidase	% Identity	% Positives	Activity[a] nmol/mg/h	% Identity	% Positives
Arabidopsis thaliana	BT028918.1 At1q77670.1	AY093711.1 At5g12040	100	100	2.8[b]	100	100
Chlamydomonas reinhardti	XM_001690029.1	XM_001690787.1	46	62	110.3	64	80
Glycine max	XM_003526572.4	XM_003525995.4	78	88	31.9	86	92
Oryza sativa	XM_015756331.1	AK059981.1	83	91	1.72	72	82
Physcomitrium patens	XP_001771268.1	XM_024542108.1	71	86	4.98	72	85

Supplemental Table S2. Estimation of assimilated N in untreated and treated plants.

This estimation of assimilated N in the plant ignores the contribution of nucleic acids, secondary metabolites, and free nitrate in the plant. Therefore, this does not replace measurement of total N by the Kjelhahl method.

Oats	Root Protein mg/gfwt	Root Weight g	Total[a] Root Protein, g	Leaf Protein mg/gfwt	Leaf Weight g	Total[b] Leaf Protein, g	Total[c] Plant Protein, g	Estimated[d] Assimilated Nitrogen[e] g/plant
Control	3.9 ±0.04	4.3 ±0.7	16.77	6.0 ±0.50	4.6 ±0.7	27.6	44.37	7.09
Treated	4.1 ±0.06	8.3 ±1.1	34.03	8.8 ±0.3	7.9 ±1.1	69.52	103.55	16.57

[a]Total Root Protein = Root Protein x Root Weight
[b]Total Leaf Protein = Leaf Protein x leaf weight
[c]Total Plant Protein = Total Root Protein + Total Leaf Protein
[d]Total Estimated Assimilated Nitrogen = Total Plant Protein x 0.16*

*Proteins have historically been assumed to be 16% nitrogen[1]. More recently the estimation is being refined to account for differences between plant species (2019)[1] . However, for our purpose of making a comparison between plant populations of the same species and variety this assumption seems reasonable.

[1]ACS.Onlinelibrary.wiley.com. Calculation of Nitrogen to Protein Conversion Factors
